# The Association of Bone-related Biomarkers With Incident Hip Fracture: A Nested Case-control Study

**DOI:** 10.1210/jendso/bvaf148

**Published:** 2025-09-10

**Authors:** Sara J Cromer, Elaine W Yu, Elisabetta Patorno, Gary C Curhan, Julie M Paik

**Affiliations:** Department of Medicine, Harvard Medical School, Boston, MA 02115, USA; Endocrine Unit, Massachusetts General Hospital, Boston, MA 02114, USA; Division of Pharmacoepidemiology and Pharmacoeconomics, Department of Medicine, Brigham and Women's Hospital, Boston, MA 02115, USA; Department of Medicine, Harvard Medical School, Boston, MA 02115, USA; Endocrine Unit, Massachusetts General Hospital, Boston, MA 02114, USA; Department of Medicine, Harvard Medical School, Boston, MA 02115, USA; Division of Pharmacoepidemiology and Pharmacoeconomics, Department of Medicine, Brigham and Women's Hospital, Boston, MA 02115, USA; Division of Epidemiology, Harvard T. H. Chan School of Public Health, Boston, MA 02115, USA; Department of Medicine, Harvard Medical School, Boston, MA 02115, USA; Division of Epidemiology, Harvard T. H. Chan School of Public Health, Boston, MA 02115, USA; Channing Division of Network Medicine, Department of Medicine, Brigham and Women's Hospital, Boston, MA 02115, USA; Division of Renal (Kidney) Medicine, Department of Medicine, Brigham and Women's Hospital, Boston, MA 02115, USA; Department of Medicine, Harvard Medical School, Boston, MA 02115, USA; Division of Pharmacoepidemiology and Pharmacoeconomics, Department of Medicine, Brigham and Women's Hospital, Boston, MA 02115, USA; Channing Division of Network Medicine, Department of Medicine, Brigham and Women's Hospital, Boston, MA 02115, USA; Division of Renal (Kidney) Medicine, Department of Medicine, Brigham and Women's Hospital, Boston, MA 02115, USA; New England Geriatric Research Education and Clinical Center, VA Boston Healthcare System, Boston, MA 02130, USA

**Keywords:** hip fracture, osteoporosis, osteocalcin, CTX, sclerostin, bicarbonate

## Abstract

**Context:**

Current osteoporosis risk stratification relies on clinical factors and bone mineral density alone.

**Objective:**

To determine if osteocalcin, c-terminal cross-linking telopeptide of type 1 collagen, sclerostin, and bicarbonate (“bone-related biomarkers”) are associated with future fracture risk or improve risk stratification.

**Design:**

Nested, matched case-control.

**Setting:**

Longitudinal cohorts of health care workers.

**Patients:**

Individuals with and without hip fracture in the Nurses’ Health Study I and the Health Professionals Follow-up Study.

**Main Outcome Measure:**

Hip fracture.

**Results:**

Among 642 women in Nurses’ Health Study I (mean age, 70.3 years; 29% with osteoporosis), we found no consistent associations between bone-related biomarkers and incident hip fracture, and addition of biomarkers to clinical models predicting incident hip fracture did not improve model fit. Among 586 men in Health Professionals Follow-up Study (mean age, 63.8 years; <1% with osteoporosis), higher levels of osteocalcin (odds ratio, 0.37 [95% CI, 0.13-1.04] for quintile 5 vs quintile 1; *P* for trend = .02) and sclerostin (odds ratio, 0.22 [95% CI, 0.09-0.54] for quintile 5 vs quintile 1; *P* for trend < .001) were associated with lower risk of hip fracture; however, addition of sclerostin to clinical models predicting incident hip fracture provided limited additional predictive value.

**Conclusion:**

Osteocalcin, c-terminal cross-linking telopeptide of type 1 collagen, sclerostin, and bicarbonate were not associated with incident hip fracture among older, predominantly White women. Osteocalcin and sclerostin were associated with hip fracture among men but did not meaningfully improve the predictive accuracy of models based on clinical risk factors alone.

Osteoporosis is a common disease, with an estimated 30% to 50% of women and 20% to 25% of men over age 50 years expected to experience an osteoporotic fracture in their lifetime [1-4]. One of the most severe consequences of osteoporosis, hip fracture, is highly morbid, with 1-year mortality rates as high as 35% to 50%, depending on the population [5, 6], and is increasing in frequency over time, at least in part due to the aging world population [5, 7].

At present, the decision to initiate highly effective medications to prevent hip fracture is based primarily on risk stratification based on clinical factors. The most important of these factors are history of prior fragility fracture and measures of bone mineral density (BMD). However, guidelines also recommend that risk stratification consider demographics (eg, age), medications (eg, glucocorticoids), comorbidities (eg, rheumatoid arthritis), and lifestyle factors (eg, smoking), which may affect bone health and BMD trajectory [8-14].

Although current guidelines recommend risk stratification based on these clinical factors, there has been interest in identifying biomarkers that may further improve risk stratification, and a number of studies of various design have demonstrated an association between certain blood biomarkers and bone health, leading some authors to suggest these biomarkers may improve fracture risk assessment [15-17]. For instance, longitudinal studies have reported variable associations of bone remodeling proteins derived from osteoblasts, osteoclasts, and osteocytes—including osteocalcin, c-terminal cross-linking telopeptide of type 1 collagen (CTX), and sclerostin—with prospective hip fracture risk [18-20]. Similarly, acidosis as marked by low bicarbonate levels has been associated with reduced bone mass, and supplementation of bicarbonate has led to improved calcium balance and BMD in small, randomized trials [21-27]. However, despite these associations, there is no conclusive evidence to suggest that these biomarkers provide additional predictive value beyond clinical risk factors when assessing long-term fracture outcomes in human populations, particularly in long-term prediction of fracture risk. Incorporation of these markers, in addition to clinical risk factors, in fracture risk algorithms has been identified as an area with interest by expert panels [28].

In this study, we performed a nested case-control analysis on participants with and without hip fracture who participated in the Nurses’ Health Study I (NHS) and Health Professionals Follow-up Study (HPFS) longitudinal cohorts. We examined the association of biomarkers implicated in bone function and health (osteocalcin [total], CTX, sclerostin, and bicarbonate; henceforth “bone-related biomarkers”) with future hip fracture over a median follow-up of more than a decade and tested whether inclusion of these biomarkers improved fracture prediction over clinical factors alone and therefore whether they may have a role in clinical risk prediction in routine care.

## Materials and Methods

### Study Design and Population

We employed a nested case-control design within the NHS and HPFS cohorts. The NHS enrolled 121 700 female nurses between the ages of 30 and 55 years in 1976, collecting demographic, survey-based, and anthropomorphic information and biologic samples (collected in approximately 33 000 individuals) at multiple time points with >90% follow-up in the first 38 years of the study to ascertain outcomes. The HPFS enrolled 51 529 male health professionals between the ages of 40 and 75 years beginning in 1986, collecting similar information with ∼90% follow-up in the first 26 years of the study. The study protocol was approved by the institutional review boards of the Brigham and Women's Hospital and the Harvard T.H. Chan School of Public Health.

Within these cohorts, we selected 614 cases who experienced hip fracture during follow-up and 614 controls who did not experience hip fracture during follow-up, matching cases and controls on age at biomarker collection (within 25 months, 90% matched within 12 months), sex, self-reported race or ethnicity (although both cohorts are >90% non-Hispanic White), menopausal status and use of postmenopausal hormones (NHS only), season, fasting status, and time of blood collection (morning vs other), and excluding those with known fragility fracture before the date of blood collection.

### Metabolite Processing

Archived plasma samples collected at the time point closest to and preceding hip fracture for cases (with matched time point for controls) were processed in a centralized laboratory with extensive experience processing plasma samples from these cohorts. Quality control procedures have been performed at multiple time points in each cohort to ensure minimal sample degradation. We measured plasma levels of osteocalcin (total) and CTX using electrochemiluminescence immunoassays (Roche E Modular system; for osteocalcin, the lowest detection limit of this assay is 0.50 ng/mL and the day-to-day imprecision values at concentrations of 7.04, 25.5, and 78.1 ng/mL are 1.6%, 1.4%, and 1.5%, respectively; for CTX, the lowest detection limit of this assay is 0.01 ng/mL and the day-to-day imprecision values at concentrations of 0.08, 0.39 and 3.59 ng/mL are 4.7%, 4.3%, and 1.6%, respectively). We measured sclerostin using a quantitative sandwich enzyme immunoassay (Alpco Diagnostics, Salem, NH; sensitivity of 8.9 pmol/L and a run-to-run imprecision at sclerostin concentrations of 13 and 35 pmol/L of 5.0% and 6.0%, respectively). Finally, we measured bicarbonate using an enzymatic technique on the Roche P Modular system (intra-assay coefficient of variability, 4.5%). Bicarbonate was not available in HPFS because of the type of preservative used for sample storage.

### Exposures and Outcomes

The primary exposures of this analysis are bone-related plasma biomarkers, including osteocalcin, CTX, sclerostin, and bicarbonate (NHS only). Although samples were collected at variable times of day and often not in the fasting state, which can affect CTX levels [29, 30], cases and controls were matched on both fasting status and time of blood collection.

Due to the nonnormal distributions of these biomarkers and desire to examine for nonlinear or threshold effects, each was transformed into quintiles (based on distributions in the control group; Table 1; Table S1) [31] and analyzed as a categorical variable. The primary outcome was self-reported hip fracture, a measure previously validated as having high accuracy, including both presence and date of fracture, in this cohort [32].

**Table 1.** Baseline characteristics of included participants from the Nurses’ Health Study I and the Health Professionals Follow-up Study, with and without hip fracture

	NHS I: All (n = 642)	NHS I: Controls (n = 321)	NH I: Cases (n = 321)	HPFS: All (n = 586)	HPFS: Controls (n = 293)	HPFS: Cases (n = 293)
Age at biomarker assessment, years*^a^*	70.3 (6.3)	70.3 (6.3)	70.3 (6.4)	63.8 (8.6)	63.8 (8.5)	63.9 (8.6)
Race or ethnicity, n (%)						
Non-Hispanic White	611 (95.2)	304 (94.8)	307 (95.6)	559 (95.4)	281 (95.9)	278 (94.9)
Other or mixed*^b^*	31 (4.8)	17 (5.2)	14 (4.4)	6 (1.0)	2 (0.7)	4 (1.3)
Missing	—	—	—	21 (3.6)	10 (3.4)	11 (3.8)
Body mass index, kg/m^2^	24.3 (22.1, 27.5)	24.9 (22.6, 28.1)	23.9 (21.6, 26.6)	25.1 (23.1, 27.4)	25.1 (23.1, 27.3)	25.1 (23.1, 27.5)
Physical activity, METs/day*^c,d^*	13.1 (5.1, 25.8)	14.3 (6.2, 25.9)	11.5 (4.3, 25.7)	27.1 (12.6, 50.9)	26.8 (10.2, 53.9)	27.7 (13.6, 49.7)
Dietary calcium intake, mg/day*^d^*	720 (574, 933)	720 (583, 917)	726 (561, 946)	766.5 (623.5, 984.5)	764 (625, 951)	767 (622, 1019)
Supplementary calcium intake, mg/day, mean (SD)*^d^*	522.2 (490.3)	491.1 (476.4)	553.9 (501.5)	120.6 (271.6)	111.3 (277)	128.6 (264)
Dietary vitamin D intake, IU/day*^d^*	162.8 (106, 253)	163.5 (110, 259)	161.5 (101, 248)	237 (165, 355)	244 (165, 355)	237 (176, 354)
Supplementary vitamin D intake, mg/day, mean (SD)*^d^*	258.3 (232.7)	252 (230.1)	263.9 (235.3)	174.6 (237.0)	171.1 (234.4)	178.5 (239.7)
Caffeine intake, mg/day*^d^*	151.3 (37.2, 322.7)	182.3 (50.8, 329.7)	127.0 (20.4, 308.1)	157.0 (43, 359)	144.6 (42, 354.5)	170 (45.6, 359.0)
Alcohol, g/day*^d^*	1.1 (0, 6.7)	1.5 (0, 6.7)	1.0 (0, 6.7)	4.9 (0.9, 15.6)	6.5 (1.0, 18.2)	3.9 (0, 13.8)
Total protein intake, g/day*^d^*	66.6 (59.4, 74.7)	66.6 (59.8, 74.5)	66.7 (59.0, 74.8)	88.3 (78.2, 98.4)	88.8 (77.7, 100.1)	88.1 (78.4, 98.1)
Animal protein intake, g/day*^d^*	43.8 (36, 51.9)	43.9 (37.1, 51.7)	43.8 (35.4, 52.6)	60.9 (50.6, 72.6)	61.2 (49.4, 72.8)	60.9 (51.5, 72.4)
Vegetable protein intake, g/day*^d^*	22.6 (19.7, 25.5)	22.2 (19.8, 25.1)	22.9 (19.6, 26.1)	26.3 (22.8, 30.7)	26.7 (23.1, 30.6)	26 (22.4, 30.7)
Dairy protein intake, g/day*^d^*	13.6 (9.2, 19.5)	14.0 (10, 19.8)	13.1 (8.4, 19.3)	14.1 (9.6, 20.9)	14.4 (9.2, 19.3)	13.7 (9.8, 21.4)
Nondairy protein intake, g/day*^d^*	28.4 (21.3, 35.6)	28.2 (21.7, 34.7)	28.4 (20.5, 36.6)	36.7 (28.3, 46.0)	38.1 (28.2, 46.2)	36.3 (28.4, 45.7)
Smoking status						
Never, n (%)	324 (50.5)	161 (50.3)	162 (50.5)	272 (46.4)	145 (49.4)	127 (43.5)
Past, n (%)	287 (44.7)	145 (45.2)	142 (44.1)	273 (46.6)	130 (44.4)	142 (48.5)
Current, n (%)	30 (4.7)	13 (4.1)	17 (5.3)	20 (3.4)	10 (3.5)	10 (3.4)
Missing, n (%)	1 (0.2)	1 (0.3)	0 (0)	21 (3.6)	8 (2.8)	13 (4.5)
History of hypertension, n (%)	326 (50.8)	168 (52.3)	158 (49.1)	197 (33.6)	98 (33.6)	98 (33.6)
History of diabetes, n (%)	48 (7.5)	26 (8.1)	22 (6.9)	33 (5.6)	11 (3.7)	22 (7.5)
History of coronary heart disease, n (%)	23 (3.6)	11 (3.4)	12 (3.8)	38 (6.5)	16 (5.4)	22 (7.4)
History of cerebrovascular accident, n (%)	22 (3.4)	7 (2.2)	15 (4.7)	6 (1.0)	3 (1.0)	3 (1.0)
History of osteoporosis, n (%)	186 (29)	81 (25.1)	105 (32.7)	5 (0.9)	2 (0.6)	3 (1.0)
History of rheumatoid arthritis, n (%)	70 (10.9)	32 (10.0)	38 (11.9)	24 (4.1)	13 (4.3)	12 (4.1)
Glucocorticoid use, n (%)	40 (6.2)	15 (4.7)	25 (7.8)	18 (3.1)	9 (3.1)	9 (3.1)
Thiazide diuretic use, n (%)	109 (17.0)	49 (15.2)	60 (18.6)	20 (3.4)	10 (3.4)	10 (3.4)
Bisphosphonate use, n (%)	103 (16.0)	57 (17.7)	46 (14.4)	30 (5.1)	8 (2.7)	22 (7.6)
History of falls, n (%)	207 (32.2)	104 (32.5)	102 (31.7)	—	—	—
Postmenopausal hormone replacement therapy use, n (%)						
Ever used	118 (18.4)	59 (18.5)	58 (18.1)	—	—	—
Past PMH use	149 (23.2)	76 (23.8)	74 (23.0)	—	—	—
Current PMH use	349 (54.4)	173 (54)	175 (54.6)	—	—	—
Missing	26 (4.0)	12 (3.7)	14 (4.4)	—	—	—
Time of blood draw, n (%)						
Morning	562 (87.5)	283 (88.1)	279 (87)	414 (70.6)	209 (71.5)	204 (69.7)
Afternoon or night	39 (6.1)	15 (4.6)	24 (7.4)	106 (18.1)	50 (17.2)	56 (19.0)
Missing	41 (6.4)	23 (7.2)	18 (5.6)	66 (11.3)	33 (11.3)	33 (11.2)
Fasting status at blood collection, n (%)						
Nonfasting	564 (87.9)	295 (92)	269 (83.8)	274 (46.8)	137 (46.9)	136 (46.5)
Fasting >8 hours	78 (12.1)	26 (8.0)	52 (16.2)	312 (53.2)	156 (53.1)	157 (53.5)
Calcium (mg/dL)	9.4 (0.4)	9.4 (0.5)	9.4 (0.4)	—	—	—
Creatinine (mg/dL)	0.8 (0.2)	0.8 (0.1)	0.8 (0.2)	0.9 (0.2)	0.9 (0.1)	0.9 (0.2)
PTH (pg/mL)	34.7 (14.0)	34.3 (12.5)	35.1 (15.3)	37.2 (17.0)	36.4 (12.5)	38 (20.0)
Vitamin D (ng/mL)	27 (8.2)	27.7 (8.3)	26.3 (8.1)	27.8 (13.2)	28.5 (16.9)	27.2 (8.2)
Osteocalcin (ng/mL)						
Full sample, median (IQR)	13.0 (10.1, 17.5)	12.9 (9.8, 17.3)	13.5 (10.3, 17.7)	21.9 (18.0, 25.8)	22.0 (18.2, 26.2)	21.7 (17.9, 25.6)
Quintile 1, median (IQR)	7.7 (6.4, 8.2)	7.7 (6.4, 8.2)	7.4 (6.3, 8.2)	15.4 (13.9, 16.5)	15.8 (14.3, 16.6)	15.1 (13.3, 16.4)
Quintile 2, median (IQR)	10.5 (9.8, 11.1)	10.3 (9.8, 11.0)	10.5 (9.9, 11.2)	19.2 (18.1, 19.9)	19.4 (18.2, 20.1)	19.1 (18.0, 19.8)
Quintile 3, median (IQR)	12.8 (12.2, 13.6)	12.9 (12.2, 13.5)	12.7 (12.2, 13.7)	22.2 (21.5, 22.9)	22.1 (21.4, 23.0)	22.3 (21.7, 22.9)
Quintile 4, median (IQR)	16.3 (15.2, 17.3)	16.1 (15.0, 17.3)	16.4 (15.2, 17.3)	25.3 (24.2, 26.2)	25.0 (24.3, 26.2)	25.6 (24.2, 26.2)
Quintile 5, median (IQR)	22.4 (20.1, 26.1)	21.7 (20.0, 24.9)	23.3 (20.6, 27.2)	31.7 (29.7, 35.8)	31.2 (29.7, 33.4)	32.9 (29.9, 37.6)
CTX (ng/mL)						
Full sample, median (IQR)	0.2 (0.1, 0.3)	0.2 (0.1, 0.3)	0.2 (0.1, 0.3)	0.3 (0.2, 0.4)	0.3 (0.2, 0.4)	0.3 (0.2, 0.4)
Quintile 1, median (IQR)	0.1 (0.1, 0.1)	0.1 (0.1, 0.1)	0.1 (0.1, 0.1)	0.2 (0.1, 0.2)	0.2 (0.1, 0.2)	0.2 (0.1, 0.2)
Quintile 2, median (IQR)	0.1 (0.1, 0.2)	0.1 (0.1, 0.2)	0.1 (0.1, 0.2)	0.2 (0.2, 0.2)	0.2 (0.2, 0.2)	0.2 (0.2, 0.3)
Quintile 3, median (IQR)	0.2 (0.2, 0.2)	0.2 (0.2, 0.2)	0.2 (0.2, 0.2)	0.3 (0.3, 0.3)	0.3 (0.3, 0.3)	0.3 (0.3, 0.3)
Quintile 4, median (IQR)	0.3 (0.3, 0.3)	0.3 (0.3, 0.3)	0.3 (0.3, 0.3)	0.4 (0.4, 0.4)	0.4 (0.4, 0.4)	0.4 (0.4, 0.4)
Quintile 5, median (IQR)	0.4 (0.4, 0.5)	0.5 (0.4, 0.5)	0.4 (0.4, 0.5)	0.5 (0.5, 0.7)	0.5 (0.5, 0.7)	0.5 (0.5, 0.7)
Sclerostin (pmol/L)						
Full sample, median (IQR)	42.4 (33.4, 52.3)	43.6 (35.1, 52.7)	41.2 (33.2, 51.8)	51.0 (40.5, 63.8)	53.3 (42.6, 69.9)	48.4 (38.6, 60.4)
Quintile 1, median (IQR)	28.2 (23.9, 30.3)	28.1 (23.9, 30.5)	28.2 (23.9, 30.1)	32.6 (28.7, 35.6)	32.5 (28.9, 35.4)	32.7 (28.3, 37.1)
Quintile 2, median (IQR)	36.0 (33.6, 38.1)	36.7 (35.1, 39.2)	35.4 (33.4, 37.6)	44.3 (42.5, 46.8)	44.1 (42.6, 46.6)	44.8 (42.1, 46.8)
Quintile 3, median (IQR)	43.7 (42.0, 45.1)	43.6 (42.0, 45.3)	44.1 (42.0, 44.9)	54.1 (51.7, 56.8)	53.3 (51.0, 56.9)	54.9 (52.4, 56.7)
Quintile 4, median (IQR)	51.0 (48.9, 52.7)	51.2 (49.3, 53.0)	50.3 (48.5, 52.7)	64.9 (61.8, 69.8)	64.8 (61.6, 69.9)	65.4 (62.1, 69.8)
Quintile 5, median (IQR)	67.0 (59.7, 76.2)	66.4 (59.9, 73.7)	67.6 (59.6, 76.4)	84.5 (81.3, 95.6)	87.2 (81.6, 97.9)	84.2 (80.0, 95.4)
Bicarbonate (mmol/L)						
Full sample, median (IQR)	21.6 (20.2, 23.3)	21.5 (20.1, 23.2)	21.8 (20.4, 23.3)	—	—	—
Quintile 1, median (IQR)	18.5 (17.5, 19.3)	18.7 (17.0, 19.4)	18.4 (17.5, 19.3)	—	—	—
Quintile 2, median (IQR)	20.5 (20.2, 20.8)	20.5 (20.1, 20.8)	20.6 (20.2, 20.9)	—	—	—
Quintile 3, median (IQR)	21.5 (21.3, 21.8)	21.5 (21.3, 21.9)	21.5 (21.3, 21.7)	—	—	—
Quintile 4, median (IQR)	22.8 (22.4, 23.3)	22.8 (22.3, 23.3)	22.7 (22.4, 23.2)	—	—	—
Quintile 5, median (IQR)	24.8 (24.2, 25.9)	24.8 (24.3, 25.9)	24.8 (24.2, 25.9)	—	—	—

Cases and controls were matched on age, sex (within cohorts), self-reported race or ethnicity, fasting time, and date and time of blood collection. Values are means (SD) or median (IQR) for continuous variables and number (%) for categorical variables, and are standardized to the age distribution of the study population. Categorical variables may not sum exactly to 100% due to rounding.

Abbreviations: CTX, c-terminal cross-linking telopeptide of type 1 collagen; IQR, interquartile range; METS, metabolic equivalents; PMH, postmenopausal hormone.

^a^Value is not age-adjusted.

^b^Includes all individuals who did not self-identify as non-Hispanic White or non-Hispanic Black.

^c^Metabolic equivalents from recreational and leisure-time activities.

^d^Data were missing for 5 and 7 individuals (physical activity) and 13 and 22 individuals (dietary and supplemental calcium, dietary and supplemental vitamin D, caffeine, alcohol, and total, animal, vegetable, dairy, and nondairy protein intake) in the Nurses’ Health Study I and Health Professionals Follow-up Study, respectively. For analyses, these individuals were assigned to a “missing” category.

Beyond the matching factors listed here, “fully adjusted” and “combined” models adjusted for clinical covariates known to associate with bone health including body mass index (BMI, kg/m^2^); self-reported physical activity (metabolic equivalents per day); smoking status; self-report of recent falls (within past 2 years; NHS only); the presence or absence of self-reported diagnoses of hypertension, diabetes, cardiovascular disease, osteoporosis, rheumatoid arthritis, and cancer; self-reported use of thiazide diuretics, glucocorticoids, and bisphosphonates; laboratory values obtained from a centralized laboratory including plasma levels of 25-OH vitamin D (ng/mL), calcium (mg/dL; NHS only), creatinine (mg/dL), and PTH (pg/mL); and dietary factors from validated food frequency questionnaires, including daily intake of: dietary and supplementary calcium (mg/day); dietary and supplementary vitamin D (international units (IU)/day); caffeine (mg/day); alcohol (g/day); animal, vegetable, and dairy protein (g/day). BMI, physical activity, and intakes of calcium, vitamin D, caffeine, alcohol, and proteins (animal, vegetable, dairy) were modeled by quintile. All covariates were assessed from the questionnaire closest to sample collection, which was within 2 years of sample collection in 94% of NHS and 98% of HPFS participants.

### Statistical Analysis

We describe baseline characteristics at the time of sample collection of cases and controls using mean and SD or median and interquartile range for parametric and nonparametric continuous variables, respectively, and numbers and percentages for categorical variables. Each was standardized to the age distribution of the study population.

We examined the association between each categorized biomarker and hip fracture in conditional logistic regression models, calculating adjusted odds ratios and 95% CIs. Initial models adjusted for the clinical and laboratory-based covariates listed previously, examining each biomarker in a different model. We next examined combined models including adjustment for clinical covariates in addition to all biomarkers in a single model. We performed trend tests for quintiles of each biomarker in the full and combined models.

We performed sensitivity analyses using Cox proportional hazards (rather than conditional logistic regression) models, with additional adjustment for matching variables, to calculate hazard ratios (HR) and 95% CIs for the association between each biomarker and time-to-hip fracture.

Differences in model fit between models using clinical risk factors only vs clinical risk factors plus biomarkers were assessed using multiple methods including the Akaike information criterion (AIC) and c-indices for Cox proportional hazards models and the AIC and likelihood ratio tests for logistic regression models. An absolute decrease in AIC of 4 or an absolute increase in c-index of 0.01 was considered a significant improvement in model fit [33].

Finally, we performed stratified analyses using both unconditional logistic regression and Cox proportional hazards models to examine associations between biomarkers and hip fractures within strata of BMI (<25 kg/m^2^ vs ≥25 kg/m^2^), osteoporosis history (NHS only because of the small sample with osteoporosis in HPFS), and bisphosphonate use (NHS only because of the small sample with bisphosphonate use in HPFS). In post hoc exploratory analyses, trend tests and biomarker-by-BMI interaction tests were examined in full and combined models for the BMI <25 kg/m^2^ vs ≥25 kg/m^2^ strata, the only strata large enough for these analyses.

All analyses were performed using SAS, version 9.4 (SAS Institute, Inc.; Cary, NC).

## Results

### Baseline Characteristics

Among 614 matched pairs of cases and controls, 321 pairs were derived from the NHS cohort (mean age at biomarker assessment, 70.3 years; 100% postmenopausal women, mean BMI, 24.3 kg/m^2^; 29% with history of osteoporosis; 16% with prior bisphosphonate use) and 293 from the HPFS cohort (mean age, 63.8 years; 100% men; mean BMI, 25.1 kg/m^2^; <1% with history of osteoporosis; 5.1% with prior bisphosphonate use; 292 pairs in the sclerostin analyses as 1 pair were excluded because of a missing value; Table 1). Both cohorts included predominantly non-Hispanic White adults. Cases and controls were well-matched on most clinical characteristics, with few exceptions, notably including numerically higher rates of osteoporosis diagnosis and glucocorticoid use and lower rates of bisphosphonate use in cases than controls in NHS. Mean ± SD follow-up time between blood collection and hip fracture incidence among cases was 8.1 ± 4.2 years in NHS and 12.4 ± 6.5 years in HPFS, with 15.4 ± 4.2 and 19.6 ± 4.5 years of follow-up among controls, respectively.

### Association Between Bone-related Biomarkers and Hip Fracture

**Figure 1.**
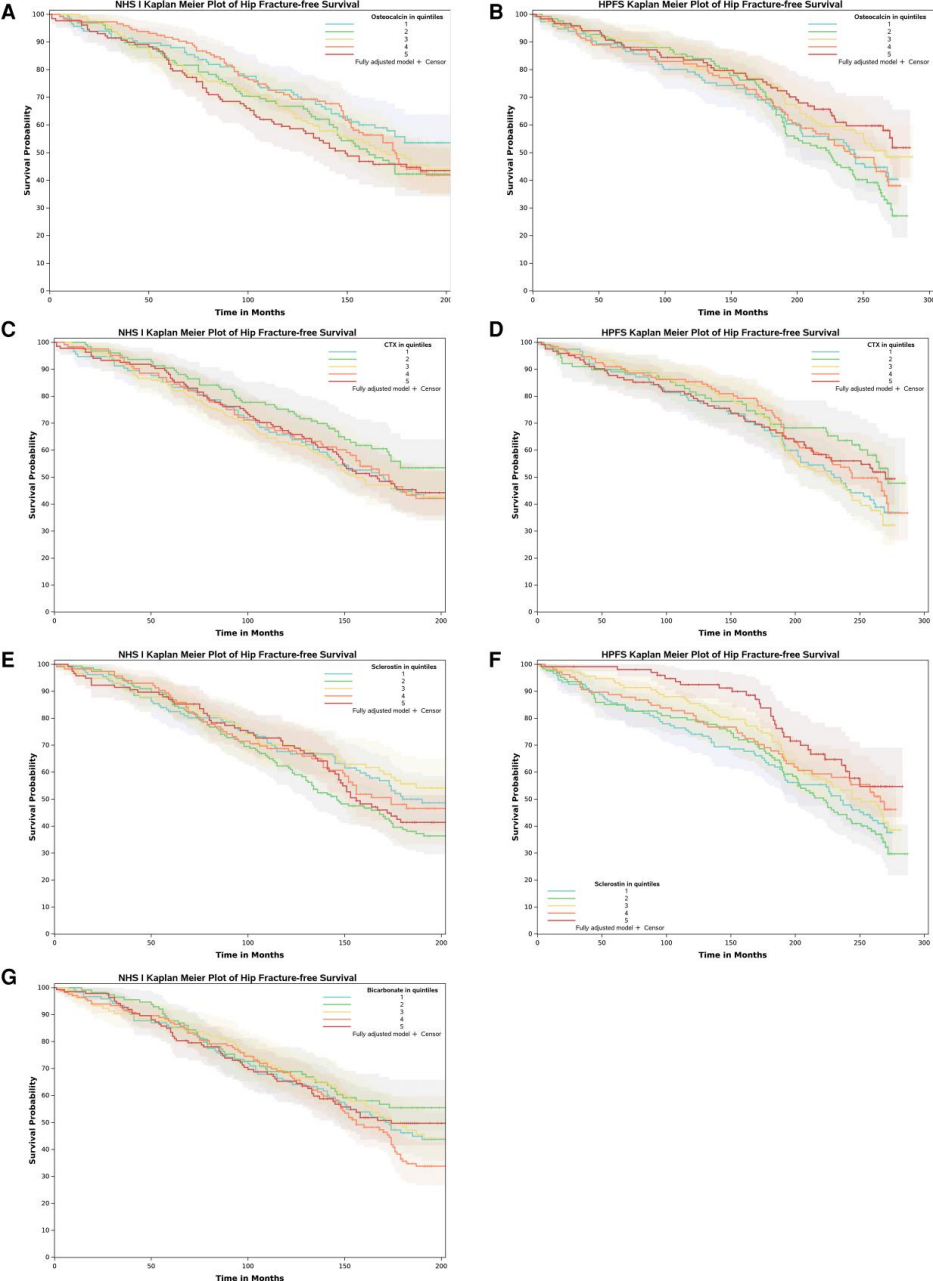
Kaplan-Meier curves for adjusted time-to-hip fracture, stratified by quintiles of (A, B) osteocalcin, (C, D) CTX, and (E, F) sclerostin in the Nurses’ Health Study (A, C, E, G) and Health Professionals Follow-up Study (B, D, F), respectively, and (G) bicarbonate (Nurses’ Health Study only).

**Figure 2.**
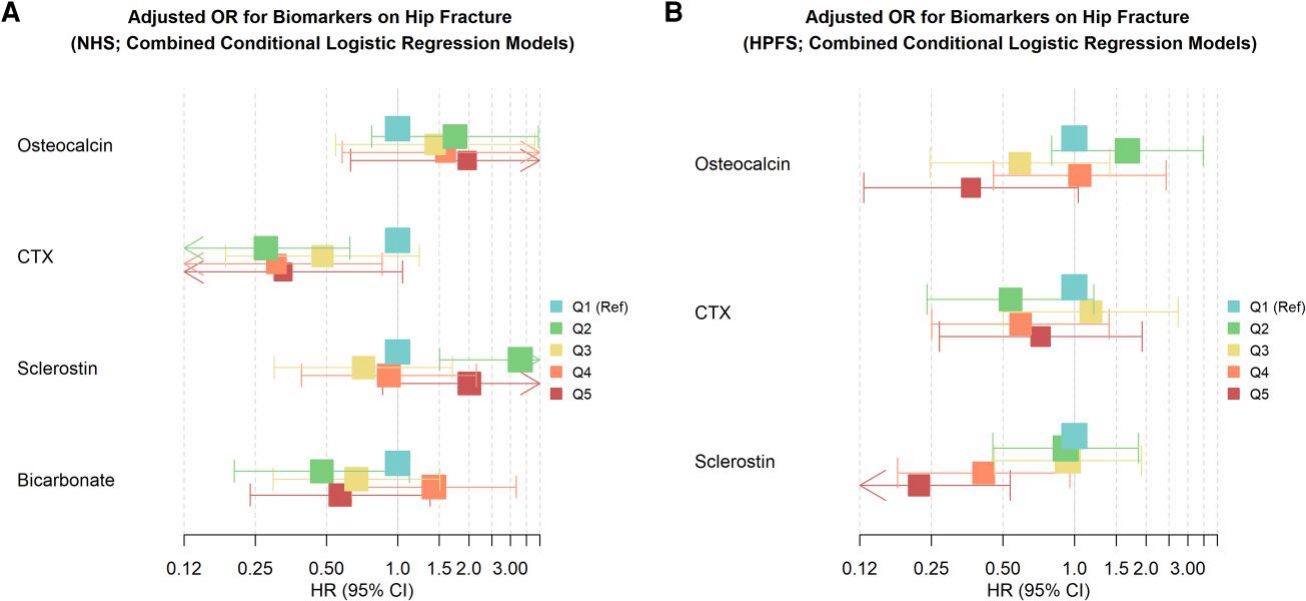
Forest plot of the association of biomarker quintiles with hip fracture in combined models adjusted for clinical covariates and including all biomarkers in (A) NHS and (B) HPFS.

Baseline, unadjusted levels of osteocalcin, CTX, sclerostin, and bicarbonate (NHS only) were similar between cases and controls in both cohorts (Table 1). In fully adjusted models (including all bone-related clinical covariates and each bone-related biomarker in a separate model) and in combined models (including all covariates and biomarkers in a single model), no single bone-related biomarker was consistently associated with hip fracture in NHS (Table 2). However, in HPFS, higher levels of osteocalcin (HRs [95% CIs] with Q1 as reference: 1.67 [0.80-3.49], 0.59 [0.25-1.41], 1.05 [0.46-2.43], and 0.37 [0.13-1.04] for quintiles 2 through 5, respectively; *P* for trend .02) and sclerostin (HRs [95% CIs] with Q1 as reference: 0.92 [0.45-1.86], 0.94 [0.46-1.91], 0.42 [0.18-0.96], and 0.22 [0.09-0.54] for quintiles 2 through 5, respectively, in combined models; *P* for trend <.001) associated with reduced risk of hip fracture (Table 2; Figs. 1 and 2). Findings were similar in sensitivity analyses examining fracture risk using Cox proportional hazards models (Table S2) [31].

**Table 2.** Association of bone-related biomarkers with hip fracture, in conditional logistic regression models adjusted only for matched factors or for all clinical fracture predictors available

	NHS I (OR, 95% CI)		HPFS (OR, 95% CI)	
	Adjusted models*^a^* (4 models)	Combined models*^b^* (4 biomarkers in one model)	Adjusted models*^a^* (4 models)	Combined models*^b^* (4 biomarkers in 1 model)
Osteocalcin				
Quintile 1 (Ref)	Ref	Ref	Ref	Ref
Quintile 2	1.55 (0.76, 3.14)	1.75 (0.78, 3.94)	1.75 (0.90, 3.40)	1.67 (0.80, 3.49)
Quintile 3	1.06 (0.47, 2.39)	1.44 (0.55, 3.80)	0.67 (0.31, 1.43)	0.59 (0.25, 1.41)
Quintile 4	1.08 (0.52, 2.25)	1.60 (0.58, 4.38)	1.33 (0.64, 2.77)	1.05 (0.46, 2.43)
Quintile 5	1.24 (0.56, 2.74)	1.97 (0.63, 6.13)	0.43 (0.19, 0.99)	0.37 (0.13, 1.04)
*P* for trend	.77	.83	**.02**	**.02**
CTX				
Quintile 1 (Ref)	Ref	Ref	Ref	Ref
Quintile 2	0.50 (0.25, 0.99)	0.28 (0.12, 0.63)	0.49 (0.23, 1.03)	0.54 (0.24, 1.21)
Quintile 3	1.03 (0.51, 2.11)	0.48 (0.19, 1.23)	1.24 (0.60, 2.57)	1.17 (0.50, 2.73)
Quintile 4	0.59 (0.28, 1.23)	0.31 (0.11, 0.86)	0.65 (0.31, 1.37)	0.59 (0.25, 1.40)
Quintile 5	0.70 (0.32, 1.55)	0.33 (0.10, 1.05)	0.49 (0.23, 1.06)	0.72 (0.27, 1.93)
*P* for trend	.96	.98	.66	.95
Sclerostin				
Quintile 1 (Ref)	Ref	Ref	Ref	Ref
Quintile 2	3.01 (1.44, 6.27)	3.30 (1.51, 7.22)	1.03 (0.54, 1.96)	0.92 (0.45, 1.86)
Quintile 3	0.78 (0.35, 1.74)	0.72 (0.30, 1.70)	1.05 (0.55, 2.02)	0.94 (0.46, 1.91)
Quintile 4	1.10 (0.50, 2.43)	0.92 (0.39, 2.15)	0.59 (0.28, 1.22)	0.42 (0.18, 0.96)
Quintile 5	2.09 (0.95, 4.59)	2.01 (0.86, 4.69)	0.32 (0.15, 0.69)	0.22 (0.09, 0.54)
*P* for trend	.99	.79	**<.001**	**<.001**
Bicarbonate				
Quintile 1 (Ref)	Ref	Ref	—	—
Quintile 2	0.62 (0.29, 1.29)	0.48 (0.20, 1.12)	—	—
Quintile 3	0.73 (0.35, 1.51)	0.67 (0.30, 1.51)	—	—
Quintile 4	1.45 (0.71, 2.98)	1.42 (0.64, 3.18)	—	—
Quintile 5	0.61 (0.28, 1.35)	0.57 (0.24, 1.37)	—	—
*P* for trend	0.34	0.84		

Bolded values indicate statistical significance at a threshold of *P* < .05.

Abbreviations: CTX, c-terminal cross-linking telopeptide of type 1 collagen; HPFS, Health Professionals Follow-up Study; NHS I, Nurses’ Health Study I; OR, odds ratio.

^a^Adjusted models are adjusted for all clinical covariates (body mass index [kg/m^2^]; self-reported physical activity [metabolic equivalents per day]; smoking status; self-report of recent falls [NHS only]; known diagnoses of hypertension, diabetes, cardiovascular disease, osteoporosis, rheumatoid arthritis, and cancer; use of thiazide diuretics, glucocorticoids, and bisphosphonates; laboratory values including plasma levels of 25-OH vitamin D [ng/mL], calcium [mg/dL; NHS only], creatinine [mg/dL], and PTH [pg/mL]; and dietary factors, including daily intake of: dietary and supplementary calcium [mg/day]; dietary and supplementary vitamin D [international units/day]; caffeine [mg/day]; alcohol [g/day]; animal, vegetable, and dairy protein [g/day]), and include a single biomarker in each model.

^b^Combined models are adjusted for all clinical covariates and include all biomarkers in a single model.

Model diagnostics, including AIC and likelihood ratio tests for logistic regression models and AIC and c-index for Cox proportional hazards models, demonstrated no improvements with the addition of biomarkers in the NHS cohort. In the HPFS cohort, inclusion of osteocalcin, CTX, or sclerostin resulted in modest but statistically significant improvements in logistic regression model fit, but inclusion only of sclerostin resulted in significant improvements in Cox proportional hazards model fit (Table 3). For both cohorts, no biomarker improved the c-index by more than 0.02, suggesting limited additional predictive value compared to clinical factors alone (Table 3).

**Table 3.** Measures of model fit in models based on clinical factors alone and after the addition of biomarkers

	NHS I				HPFS			
	AIC (conditional logistic)	LRT *P*-value vs clinical model (logistic)	AIC (Cox)	C-index (Cox)	AIC (conditional logistic)	LRT *P*-value vs clinical model (logistic)	AIC (Cox)	C-index (Cox)
Clinical model*^a^*	467.123	—	3876.99	0.643	453.926	—	3454.854	0.632
Clinical model + osteocalcin	474.686	.79	3878.66	0.643	**443.768**	**.001**	3453.531	0.634
Clinical model + CTX	470.402	.24	3878.99	0.643	**449.579**	**.02**	3456.808	0.632
Clinical model + sclerostin	**458.830**	**.003**	3878.99	0.643	**448.621**	**.01**	**3441.401**	**0.646**
Clinical model + bicarbonate	466.593	.07	3878.26	0.643	—	—	—	—
Clinical model + all biomarkers	465.061	**.003**	3883.80	0.643	**435.973**	**<.001**	**3438.339**	**0.652**

Bolded values indicate a significant change in model performance, as defined by an absolute decrease of 4 in AIC, an absolute increase of 0.01 in c-index, and a likelihood ratio test *P* value <.05.

Abbreviations: AIC, Akaike information coefficient; CTX, c-terminal cross-linking telopeptide of type 1 collagen; HPFS, Health Professionals Follow-up Study; LRT, likelihood ratio test; NHS I, Nurses’ Health Study I.

^a^Adjusted models are adjusted for all clinical covariates (BMI [kg/m^2^]; self-reported physical activity [metabolic equivalents per day]; smoking status; self-report of recent falls [NHS only]; known diagnoses of hypertension, diabetes, cardiovascular disease, osteoporosis, rheumatoid arthritis, and cancer; use of thiazide diuretics, glucocorticoids, and bisphosphonates; laboratory values including plasma levels of 25-OH vitamin D [ng/mL], calcium [mg/dL; NHS only], creatinine [mg/dL], and PTH [pg/mL]; and dietary factors, including daily intake of: dietary and supplementary calcium [mg/day]; dietary and supplementary vitamin D [international units/day]; caffeine [mg/day]; alcohol [g/day]; animal, vegetable, and dairy protein [g/day]), and include a single biomarker in each model.

### Stratified Analyses by BMI, Osteoporosis Diagnosis, and Bisphosphonate Use

In stratified analyses by BMI, there were trends to suggest differences in the association of some biomarkers with future hip fracture in strata of individuals with BMI <25 kg/m^2^ vs BMI ≥25 kg/m^2^. For example, higher sclerostin levels seemed to be associated with higher fracture risk in women with lower BMI but with neutral or even reduced fracture risk in women with higher BMI (for stratified models, see Tables S3 and 4 [31]; *P* for interaction .067 and .057 in full and combined logistic regression models, respectively, and .039 and .034 in full and combined Cox models, respectively; interaction models not shown). In men, weak trends were seen suggesting an association between higher osteocalcin levels and lower fracture risk in the cohort overall (*P* = .066 and *P* = .016 in conditional logistic and Cox regression models, respectively); yet, in stratified analyses, this trend was only observed in the BMI <25 kg/m^2^ stratum (for stratified models, see Tables S3 and S4 [31]; *P* for interaction .069 in full models but .250 in combined logistic regression models, respectively, and .050 and .090 in full and combined Cox models, respectively; interaction models not shown). By contrast, sclerostin appeared protective in both BMI strata in logistic regression models but only in those with BMI ≥25 kg/m^2^ in Cox regression models (*P* for interaction, .182 and .027 in full and combined models, respectively; interaction models not shown). However, as these associations were underpowered and were not observed consistently across models or across quintiles, they are considered exploratory.

Within NHS only, in stratified analyses by osteoporosis diagnosis and bisphosphonate use, higher quintiles of osteocalcin were intermittently associated with increased fracture risk among individuals with osteoporosis history (Tables S3 and S4) [31]; however, fully adjusted and combined models failed to converge for some quintiles due to limited sample size in this group. Again, these findings are considered exploratory. Sample size prevented reporting of fully adjusted models in the subgroup with bisphosphonate use, and models adjusted only for matching variables are reported in Tables S3 and S4 [31]. Similar to the primary analysis, addition of biomarkers did not improve model performance for the subgroup without bisphosphonate use (Table S5) [31]. Subgroups with and without osteoporosis and with and without bisphosphonate use were not examined in HPFS due to very low rates of reported osteoporosis and bisphosphonate use at study entry.

## Discussion

In this study, we found that bone-related biomarkers, including osteocalcin, CTX, sclerostin, and bicarbonate, did not associate with incident hip fracture outcomes over an average of more than a decade of follow-up in women in a hip fracture nested case-control study, although higher sclerostin levels, and to a lesser degree higher osteocalcin levels, associated with lower fracture risk in men. The addition of biomarkers provided very limited improvement in prediction of hip fracture outcomes compared to clinical factors alone in this sample with overall low rates of osteoporosis medication use.

Osteocalcin is a protein secreted by osteoblasts and is the most common noncollagenous protein in bone [34]. Although osteocalcin is classically considered a bone formation marker and therefore may be depressed in low bone formation states (eg, glucocorticoid use), levels may also be elevated in high turnover states due to coupling of osteoblast and osteoclast activity (eg, aromatase inhibitor use). Possibly for this reason, studies have reported both higher and lower osteocalcin levels in people with osteoporosis, with a meta-analysis finding no significant difference between those with and without postmenopausal osteoporosis [35].

CTX is produced during breakdown of collagen, particularly in bone, and is considered a marker of bone turnover. Both osteocalcin and CTX levels peak 6 to 10 years after menopause [36]. Higher levels of CTX have been associated with osteoporosis and low bone density in cross-sectional studies [37, 38], and it has been shown to associate with predicted fracture risk from the FRAX tool and mortality after hip fracture but not with secondary fracture risk [19, 39, 40]. The association between both osteocalcin and CTX and subsequent fracture is variable in longitudinal studies, with some showing near-linear, dose-dependent risks with rising levels of osteocalcin or CTX and others showing no association or even u-shaped risk curves [18, 39, 41-44].

Sclerostin is secreted by osteocytes and suppresses osteoblast development and activity by antagonizing the Wnt/β-catenin pathway [45]. Circulating sclerostin levels increase with age in both men and women and are notably higher in men [46, 47], an association which may be driven by adiposity, sex hormone action, differences in skeletal mass, or by android vs gynoid fat patterns [47, 48]. Several studies have demonstrated an association between higher sclerostin levels and higher BMD in both women and men [49, 50]. However, similar to osteocalcin and CTX, longitudinal studies have found variable associations between sclerostin levels and prospective fracture risk, including higher risk or no association with fracture risk in post-menopausal women [20, 51, 52], and paradoxically lower fracture risk in older men [50, 53, 54], as seen in our study. These findings suggest that sclerostin may associate differently with future fracture risk in men and women, and further studies, notably stratified by biological sex, are needed to better understand these associations. These findings are also counterintuitive given that treatment with romosozumab, a sclerostin inhibitor which decreases circulating levels of sclerostin, is known to improve BMD in both post-menopausal women and older men [55, 56], suggesting circulating sclerostin levels may not fully reflect intracellular processes at the level of the bone.

Proposed reasons for the variability in findings from different longitudinal cohorts include differences in sample collection or processing (eg, timing of collection, inter-assay differences), variation in the exact biomarker measured (eg, blood or urine measures; total vs undercarboxylated osteocalcin—our study relied on total osteocalcin levels), and heterogeneity of how these variables were transformed and analyzed (eg, binarized, categorized in quintiles), and true associations between these biomarkers and fracture remain in debate. Overall, authors of systematic reviews and meta-analyses have concluded there is little conclusive evidence to support the use of these biomarkers in diagnosis or risk assessment for osteoporosis [34, 35, 37, 45, 57], a conclusion consistent with our findings with the possible exception of sclerostin in men.

Acidosis or acidemia, as proxied by plasma bicarbonate levels, has been implicated in bone health by a number of studies. Low bicarbonate levels have been associated with lower cross-sectional bone density, as well as more rapid decline in bone density in national health surveys and longitudinal studies of bone health [58, 59]. Several small clinical trials have also suggested that supplementation of alkaline substances, particularly potassium bicarbonate, may reduce risk factors for bone loss, including reducing urinary calcium excretion, parathyroid hormone levels, and bone turnover markers including CTX [21-25]; increasing osteocalcin [26]; and even improving BMD and architecture in some studies [27]. However, our findings suggest that bicarbonate levels did not associate with hip fracture—the most severe and clinically relevant outcome of low bone density. It is possible that the cross-sectional association between serum alkalinity and bone health may reflect confounding or even reverse causation. Alternatively, the degree of alkalinity achieved by supplementation in trials may not be reflected in the spectrum of normal diets in the NHS and HPFS cohorts, such that the level of bicarbonate needed to achieve improved bone health is not achieved without clinical intervention.

Importantly, levels of these biomarkers are known to be influenced by bisphosphonates and other osteoporosis therapies. However, we did not see any consistent effect modification in analyses stratified by bisphosphonate use, although the sample size was small, limiting our ability to adjust for likely confounders, nor did addition of biomarkers improve model performance in the stratum without bisphosphonate use. Other current osteoporosis therapies were unavailable at the time of sample collection in our cohorts and therefore would not affect our results. Biomarker levels may also be affected by recent fracture [60, 61], but this was not a factor in our selected population with biomarkers measured years prior to the time of fracture. Lastly, these measures may be affected by demographic and clinical factors, time of day at which samples were collected, fasting status (particularly for CTX), and elements of sample processing. In our analyses, these potential confounders were addressed through matching and statistical adjustment and by processing all samples in a single lab using the same assays and ensuring high levels of quality control, validity, and reliability.

Our study supports current guidelines from generalist and specialist societies regarding risk stratification and treatment of osteoporosis. These guidelines recommend treatment for fracture prevention based on a detailed assessment of clinical and biochemical risk factors which do not include the four biomarkers examined in this study [8-12]. In cases where osteoporosis is not clearly diagnosed either by dual-energy X-ray absorptiometry or by prevalent fragility fracture, risk stratification is based on tools such as the FRAX calculator which rely on anthropomorphic and other clinical fractures such as medical diagnoses and glucocorticoid use [13, 14, 62]. Our findings support this practice and additionally argue that the biomarkers tested, at least when measured cross-sectionally in a population without prior hip fracture, provide little to no additional predictive information regarding future fracture risk, with the possible exception of sclerostin in men. While we found that sclerostin levels did associate with future fracture risk in men, the addition of sclerostin to models of future fracture risk provided minimal additional predictive value beyond clinical factors alone. Although available risk calculators have a number of known limitations [62, 63], our study does not suggest that plasma biomarkers would meaningfully improve the predictive value of these algorithms in the general population. Importantly, no model in our study—with or without biomarkers, in men or in women—had a c-index greater than 0.65, suggesting overall low predictive ability even in cohorts with detailed risk factor assessment.

This study has several strengths, including its nested case-control design in a well-phenotyped cohort with extensive longitudinal follow-up, high retention rates, and the ability to adjust for many lifestyle and clinical risk factors for fracture, including dietary and laboratory variables which are often not available in other studies or even in clinical care. However, this study also has several limitations. First, biomarkers were available only in a subset of each large cohort, thus limiting sample size; this was especially of concern in the subgroups with diagnosed osteoporosis and bisphosphonate use, and our findings in these subgroups should therefore be considered exploratory, particularly given the large number of covariates included in the fully adjusted and combined models. A majority of the NHS cohort and half of the HPFS cohort were not fasting at the time of blood collection which can affect CTX levels; however, cases and controls were matched on fasting status and time of blood collection, and both factors were included as covariates in regression models. Our measure of osteocalcin was total osteocalcin, which has been less consistently associated with fracture outcomes than some related measures (carboxylated or undercarboxylated osteocalcin, or ratios of these to total osteocalcin). One of the biomarkers, bicarbonate, was only available in one of the two cohorts due to factors related to sample storage. Participants were enrolled in the 1970-1980s, almost all participants identified as non-Hispanic White, and cases and controls were selected based on completeness of data, so our findings may not reflect all contemporary dietary patterns and medications and may not be generalizable to other racial or ethnic groups with different underlying risk for fracture [64-68]. Finally, one of the strongest predictors of future fracture, BMD was not available in our sample, so we were not able to adjust for BMD or to examine associations between biomarkers and fracture in groups stratified by baseline BMD. This limitation is especially relevant in the NHS cohort as BMD screening is routinely recommended for women aged 65 years and older in the United States and therefore would likely be both available and routinely used in clinical reasoning and fracture risk calculators. As BMD was not available in our study, the predictive value of the tested biomarkers in addition to BMD cannot be assessed with our results.

In conclusion, we found that bone-related biomarkers did not associate with future hip fracture risk, with the exceptions of sclerostin and possibly osteocalcin in men, and addition of biomarkers to clinical risk prediction models did not improve or minimally improved prediction of fracture outcomes. These results support the ongoing use of primarily clinical factors in long-term fracture risk prediction and stratification models, as well as the ongoing selection of initial osteoporosis treatment course based on clinical factors alone.

## Data Availability

Restrictions apply to the availability of some or all data generated or analyzed during this study to preserve patient confidentiality or because they were used under license. The corresponding author will on request detail the restrictions and any conditions under which access to some data may be provided.

## Abbreviations

AIC: Akaike information coefficient
BMD: bone mineral density
BMI: body mass index
CTX: c-terminal cross-linking telopeptide of type 1 collagen
HPFS: Health Professionals Follow-up Study
HR: hazard ratio
NHS I: Nurses’ Health Study I
